# Acute Pancreatitis Secondary to Hemobilia after Percutaneous Liver Biopsy: A Rare Complication of a Common Procedure, Presenting in an Atypical Fashion

**DOI:** 10.1155/2018/1284610

**Published:** 2018-09-02

**Authors:** Ramy Mansour, Justin Miller

**Affiliations:** Genesys Regional Medical Center Gastroenterology, 1 Genesys Parkway, ATTN: Medical Education, Grand Blanc, MI 48439, USA

## Abstract

Percutaneous Liver Biopsy is an often-required procedure for the evaluation of multiple liver diseases. The complications are rare but well reported. Here we present a case of a 60-year-old overweight female who underwent liver biopsy for elevated alkaline phosphatase. She developed acute pancreatitis secondary to hemobilia, with atypical signs and symptoms, following the biopsy. She never had the classic triad of RUQ pain, jaundice, and upper GI hemorrhage. There were also multiple negative imaging studies, thus complicating the presentation. She was successfully treated with ERCP, sphincterotomy, balloon sweep, and stent placement. Angiography and transcatheter embolization were not required.

## 1. Introduction

Histological assessment of the liver is the gold standard for many liver and biliary disorders. Percutaneous liver biopsy (PLB) is the most commonly utilized method of obtaining tissue for analysis. Image guidance is preferred, though not mandatory, by current guidelines [1]. The complications are rare but well reported and include pain, hematoma, pneumothorax, hemobilia, peritonitis, sepsis, abscess, and death [2]. Here we present a case of one rare complication, with atypical signs and symptoms, following an image guided PLB.

## 2. Case Report

We present a case of a 60-year-old overweight female with a past medical history of type 2 diabetes, hypothyroidism, hyperlipidemia, hypertension, and internal hemorrhoids presented to her primary care physician for a health maintenance exam. She does not drink alcohol, is a lifelong nonsmoker, and denies illicit drug use. Past surgeries included cholecystectomy and total abdominal hysterectomy. She does not take antiplatelet and anticoagulant medications. She complained of hematuria during the review of systems. A CT scan was performed without contrast for the hematuria and revealed diffuse hepatic steatosis. A follow-up MRI Liver with Gadavist revealed hepatosplenomegaly with hepatic steatosis with no evidence of liver masses. Upon further discussion, she revealed complaints of pruritus. Labs were drawn after the imaging and revealed the following: AST 48 U/L (10-35 U/L), ALT 46 U/L (6-29 U/L), alkaline phosphatase 333 U/L (33-130 U/L), total bilirubin 0.4 mg/dl (0.2-1.2 mg/dl), and GGT 861 U/L (3-70 U/L) (Table 1).

She was ultimately referred to a gastroenterologist and further serologic testing was performed. She had a negative viral hepatitis panel, smooth muscle antibody, immunoglobulin-G, anti-Mitochondrial Antibody (AMA), iron saturation, ferritin, alpha-one antitrypsin genotype, and ceruloplasmin. She was a nondrinker. She had metabolic risk factors for nonalcoholic fatty liver disease but the elevated alkaline phosphatase prompted further work-up. She underwent subcostal CT guided core liver biopsy with a 17-gauge guide needle and an 18-gauge core biopsy needle inserted coaxially. Four total passes were made and ranged in size from 0.6 to 1.5 cm. The patient tolerated the procedure well. The liver biopsy revealed abundant central zone macrovesicular steatosis and a large amount of ballooning hepatocytes with Mallory-Denk bodies, consistent with steatohepatitis with fibrosis. Elevations in alkaline phosphatase can be seen in up to 1/3 of patients with nonalcoholic steatohepatitis [3], as was the case in this patient.

Two days after the biopsy, she began experiencing intermittent bouts of acute chest and epigastric abdominal pain with nausea, nonbloody emesis, and increasing stool frequency. She had several visits to the emergency department and was found to have worsening alkaline phosphatase and bilirubin levels. On postbiopsy day twelve, she had an elevated lipase of 1002 U/L (normal 20-50 U/L), but MRI/MRCP failed to reveal any abnormalities, though she was pain-free at the time of the study. The pain continued intermittently but would resolve spontaneously, with work-ups and imaging modalities (US and CT imaging) revealing no defining etiology. Seventeen days status after liver biopsy, she presented again with acute onset of epigastric pain, sharp, severe, nonradiating, and associated with nonbloody emesis, and no melena. She was found to have an elevated alkaline phosphatase and bilirubin once again but also a lipase of 2235 U/L (normal 25-50 U/L). Contrasted CT scan, ordered urgently and performed while the pain was present, finally showed pancreatitis with hyperdense material within the common bile duct.

**Figure 1:**
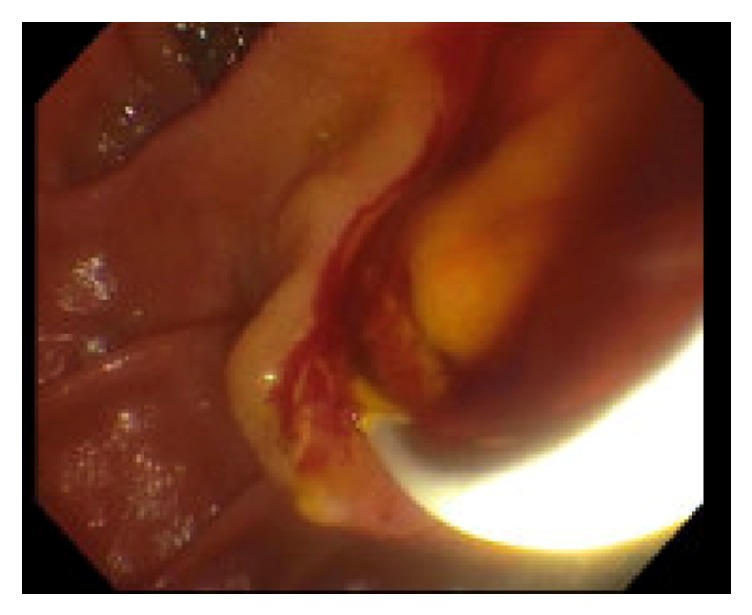
Blood coming from CBD on ERCP.

**Figure 2:**
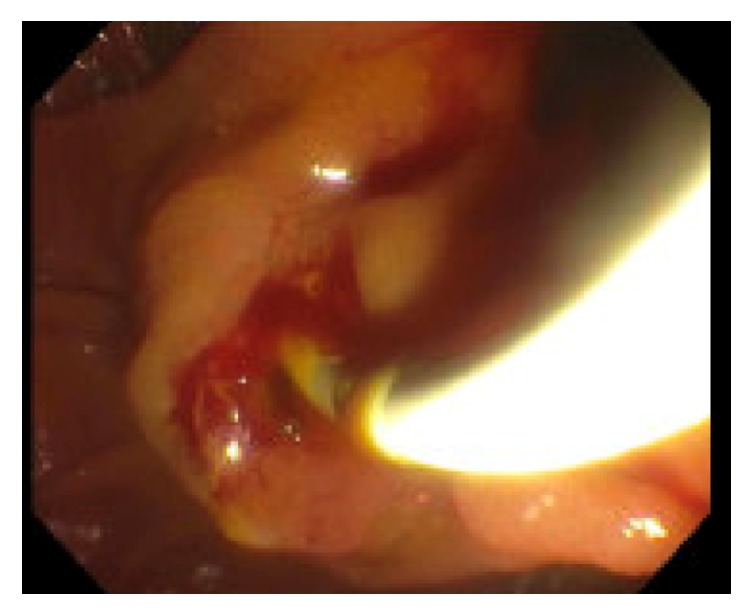
Blood clot being removed from ampulla during ERCP.

**Figure 3:**
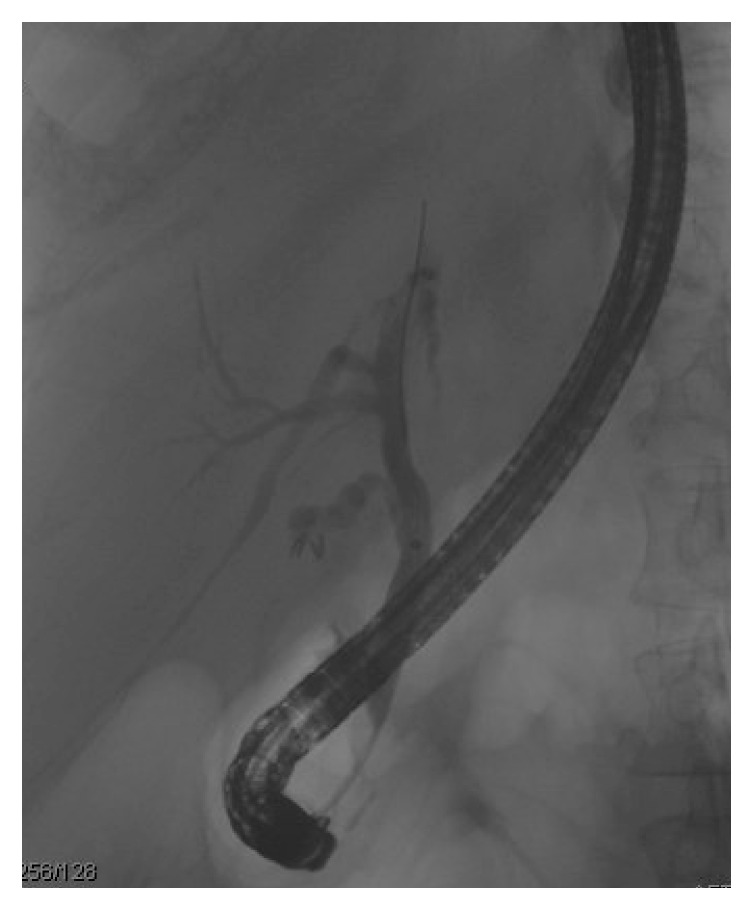
ERCP Fluoroscopy showing biliary tree with no abnormalities. However when the left intrahepatic duct was swept with a 9-12 balloon, a small amount of blood and a blood clot was evacuated.

She was referred to a tertiary center for further management. She initially underwent EGD that failed to reveal any abnormalities, including any signs of bleeding. She subsequently underwent ERCP with sphincterotomy and 9-12 mm balloon sweep of small blood clot from the left intrahepatic duct (Figures 1, 2, and 3). A 10 French by 7 cm stent was placed in the common bile duct and there was adequate bile flow. There was no evidence of further bleeding and no need for embolization. She had no further bouts of abdominal complaints.

A follow-up ERCP was performed 8 weeks later and a 9-12 mm balloon sweep with contrast was performed that did not reveal any biliary dilation or obstruction. The stent was removed successfully. Biliary drainage was noted to be adequate after procedure. Her alkaline phosphatase and bilirubin returned to their baseline prior to the liver biopsy (Table 1). She did not have any recurrence or relapse of her symptoms. There were no further signs of bleeding and her hemoglobin returned to baseline.

## 3. Discussion

Hemobilia was first theorized in 1654 by Frances Glisson on postmortem examination of a man who was stabbed by a sword in the liver and died from a GI hemorrhage [4]. It was seen throughout the years and eventually recognized antimortem. The classic triad of right upper quadrant (RUQ) pain, jaundice, and upper GI hemorrhage was first described in 1871 by Quincke [5]. Hemobilia had a grave prognosis, as there were limited treatment options. Several surgical approaches were attempted, but most were unsuccessful. In 1976, Walter was the first at successfully treating hemobilia with transcather embolization, and this has become the standard of therapy for treatment ever since [3]. However, its utility is limited to the amount of bleeding, requiring a bleeding rate of at least 0.5 to 1.0 ml/min.

Hemobilia as a complication of PLB was first reported in 1967 [6]. In a large case series of reported complications of liver biopsy, only 4 out of 68,276 patients had hemobilia [2]. The average time between liver biopsy and hemobilia onset is 5 days [7]. Several studies have described acute pancreatitis secondary to biliary obstruction from hemobilia and clot formation [8–19], the first of which was in 1975 [20]. The first cases of ERCP with sphincterotomy for treatment of acute pancreatitis secondary to hemobilia after PLB were in 1999 [11, 12]. Only three other cases since then have utilized ERCP for diagnosis and therapy of this rare adverse event [8, 9, 18].

The number of passes required during liver biopsy has been associated with a higher complication rate [1, 21, 22]. In one study by Perrault et al., the rate of complication was statistically significant when a biopsy was completed with more than 4 passes in a transthoracic approach, but not with the subcostal approach [22]. The subcostal approach was used in this patient.

## 4. Conclusion

A high index of suspicion is required to make this diagnosis. The classic triad of RUQ pain, jaundice, and upper GI hemorrhage was not observed in this case, thus delaying the diagnosis. Also, the timing of the MRI, ultrasounds, and CT scans played a role in the eventual diagnosis. The patient had intermittent symptoms and there was no evidence of acute pathology when the patient had imaging when the pain was not present. We theorize that this was likely due to spontaneous passage of the clot through the ampulla. The dilation and debris seen in the bile ducts and inflammatory changes seen in the pancreas were only noted when the patient was having severe pain at the time of imaging. There was also minimal evidence of bleeding (melena, hematochezia, decrease in hemoglobin, or elevation in BUN) until the diagnosis was already established by imaging and the patient was already scheduled for ERCP. During the ERCP, there were no abnormalities noted on fluoroscopy (Figure 3). However, since the suspected diagnosis was hemobilia with clot, the ducts were swept and ultimately revealed blood clot within the left intrahepatic biliary system (Figure 2).

Patients who suffer this rare complication of PLB historically underwent transcatheter embolization for treatment. In the case of this patient, embolization would likely not have been successful, as the patient had limited signs of bleeding and may have not been brisk enough to be picked up on standard angiography. ERCP with sphincterotomy and stent placement should be considered as an adequate adjunct treatment option, as this is the fourth case report that suggests success with ERCP treatment alone. It could also be suggested that a balloon sweep with sphincterotomy and stent placement should be performed when the diagnosis is suspected, even if this is not confirmed with cross sectional imaging or fluoroscopy at the time of ERCP.

## Figures and Tables

**Table 1:** Laboratory values before and after biopsy and ERCP.

Lab	Pre-Biopsy	7 Days Post-Biopsy	12 Days Post-Biopsy	17 Days Post-Biopsy	18 Days Post-Biopsy (Day of ERCP)	2 Months Post-ERCP
Alk Phos (U/L)	262	687	1186	889	850	280
Bilirubin (g/dL)	0.5	1.2	4.8	3.1	3.2	0.7
Lipase (U/L)			1002	2235		
AST (U/L)	32	173	273	87	152	26
ALT (U/L)	24	87	220	75	103	18
Hgb (g/dL)	11.3	12.0	11.2	10.1	7.5	10.7
